# Metformin use and hospital attendance‐related resources utilization among diabetic patients with prostate cancer on androgen deprivation therapy: A population‐based cohort study

**DOI:** 10.1002/cam4.5651

**Published:** 2023-02-03

**Authors:** Yan Hiu Athena Lee, Jeremy Man Ho Hui, Cheuk To Chung, Kang Liu, Edward Christopher Dee, Kenrick Ng, Gary Tse, Jeffrey Shi Kai Chan, Chi Fai Ng

**Affiliations:** ^1^ Division of Urology, Department of Surgery, Faculty of Medicine The Chinese University of Hong Kong Hong Kong China; ^2^ Cardio‐oncology Research Unit Cardiovascular Analytics Group, China‐UK Collaboration Hong Kong China; ^3^ Department of Radiation Oncology Memorial Sloan Kettering Cancer Center New York New York United States; ^4^ Department of Medical Oncology University College London Hospitals NHS Foundation Trust London United Kingdom; ^5^ Tianjin Key Laboratory of Ionic‐Molecular Function of Cardiovascular Disease, Department of Cardiology Tianjin Institute of Cardiology, Second Hospital of Tianjin Medical University Tianjin China; ^6^ Kent and Medway Medical School University of Kent Canterbury United Kingdom; ^7^ School of Nursing and Health Studies Hong Kong Metropolitan University Hong Kong China; ^8^ SH Ho Urology Centre, The Chinese University of Hong Kong Hong Kong China

**Keywords:** androgen deprivation therapy, diabetes, medical costs, metformin, prostate cancer

## Abstract

**Background:**

Androgen deprivation therapy (ADT), used increasingly in the treatment of prostate cancer (PCa), negatively influences glycemic control in diabetes and is associated with an increased risk of diabetes complications where hospitalization commonly ensues. Metformin could decrease the metabolic consequences of ADT and enhance its effect. This study examined the association of metformin use with healthcare resources utilization among diabetic, PCa patients receiving ADT.

**Methods:**

Diabetic adults with PCa on ADT in Hong Kong between December 1999 and March 2021 were identified. Patients with <6 months of concurrent metformin and ADT use were excluded. All included patients were followed up until September 2021. The outcomes were hospital attendances and related costs.

**Results:**

In total, 1,284 metformin users and 687 non‐users were studied. Over 8,045 person‐years, 9,049 accident and emergency (A&E), and 21,262 inpatient attendances, with 11,2781 days of hospitalization were observed. Metformin users had significantly fewer A&E attendances (incidence rate ratio (IRR): 0.61 [95% confidence interval 0.54–0.69], *p* < 0.001), inpatient attendances (IRR: 0.57 [0.48–0.67], *p* < 0.001), and days of hospitalization (IRR: 0.55 [0.42–0.72], *p* < 0.001). Annual attendance costs were lower for metformin users than non‐users (cost ratio: 0.28 [0.10–0.80], *p* = 0.017).

**Conclusions:**

Metformin use was associated with decreased hospital attendances, days of hospitalization, and associated costs, which could help reduce healthcare resource utilization following ADT in the treatment of PCa.

## Graphical abstract

Metformin users showed lower rates of Accident & Emergency and Inpatient attendances, shorter inpatient length‐of‐stay and lower attendance‐related costs compared to metformin non‐users.

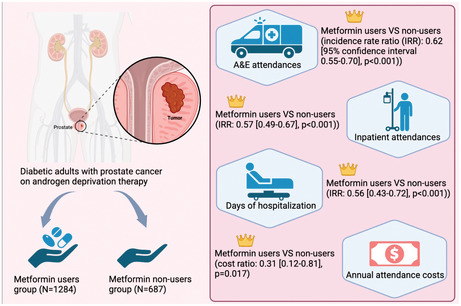

In prostate cancer (PCa) patients, androgen deprivation therapy (ADT) is the landmark treatment and is usually continued until evidence of treatment resistance in advanced and metastatic disease.^1^ While the use of ADT has been on the rise in recent years, evidence shows that it may increase the risk of adverse events where hospitalization commonly ensues.^2^, ^3^ Nonetheless, most cost and healthcare resources analyses in PCa are related to other primary treatments of PCa.^4^

In diabetic patients with PCa, metformin may reduce the metabolic side effects incurred by ADT as well as promote its efficacy.^5^ Therefore, metformin used in conjunction with ADT may lower the rate of hospitalization and its related costs. This may help alleviate the economic burden of PCa, which is an increasingly important concern in Asia where its incidence is rising.^6^ We have previously shown that metformin use in patients with PCa receiving ADT was associated with significantly lower risks of all‐cause and PCa‐related mortality,^7^ but the healthcare resources and economic implications of such associations has remained unknown. This study thus compared the rates of hospital attendances and the associated costs between metformin users and non‐users among Asian, diabetic adults with PCa receiving ADT.

This study has been ethically approved by the relevant institutional review board. Patients ≥18 years old with PCa and diabetes mellitus (DM) receiving ADT in Hong Kong between 1 December 1999 and 31 March 2021 were included. Diagnosis of PCa was identified by *International Classification of Diseases, Ninth Revision* (ICD‐9) codes (Table S1), while that of DM was determined by the corresponding ICD‐9 codes, use of antidiabetic medications at baseline, or HbA1c level >6.5% prior to ADT initiation. ADT included bilateral orchidectomy (BO) and chemical castration (gonadotropin releasing hormone agonists or gonadotropin releasing hormone antagonists).

Patients with <6 months of chemical castration without subsequent BO, <6 months of concurrent metformin and ADT use, or missing baseline HbA1c value were excluded. Metformin users had ≥6 months of concurrent metformin and ADT usage, while non‐users had no concurrent usage of metformin and ADT or never used metformin.

Continuous variables were expressed as mean ± standard deviation. To balance covariates between metformin users and non‐users, inverse treatment probability weighting (IPTW) using demographics, comorbidities, medications use, type of ADT, and PCa treatment was performed with a generalized boosted model with a maximum of 10,000 regression trees, with an iteration stopping point that minimized the maximal absolute standardized mean difference in effect size between groups. Standardized mean difference (SMD) was used to reflect inter‐group balance of covariates, with SMD values ≤0.1 considered to reflect good balance. Negative binomial regression with IPTW using follow‐up duration as an exposure variable was applied to estimate incidence rates (IR) of each group and incidence rate ratios (IRR) between groups for accident and emergency (A&E) and inpatient attendances, with 95% confidence intervals (CI). The annualized length of hospitalization and its between‐group ratio was also estimated using negative binomial regression as detailed above. A&E and inpatient attendance costs were calculated by multiplying the number of attendances with the per‐attendance cost (€148.7), and the length of hospitalization with the daily hospitalization cost (€616.5) as published by the Hong Kong Hospital Authority in 2020.^8^ The total cost related to hospital attendances was defined as the sum of A&E and inpatient attendance costs. A cost ratio with 95% CI was estimated for the total cost related to hospital attendances by using a generalized linear model with IPTW, log link, gamma distribution, and robust standard errors. The annual cost related to hospital attendances was also estimated for each group using the same generalized linear model as above. A sensitivity analysis was performed where all comparative analyses were performed using multivariable adjustments instead of IPTW. All *p* values were two‐sided, and *p* < 0.05 was considered as statistically significant. The statistical analyses were done using Stata v16.1.

In total, 2,886 patients were identified as eligible for inclusion. After applying the exclusion criteria, 1,971 patients (age 76.2 ± 7.8 years and follow‐up duration 4.1 ± 3.2 years) were analyzed (Figure S1), of whom 1,284 were metformin users, and 687 were metformin non‐users. The baseline characteristics of included patients were summarized (Table 1), and IPTW achieved satisfactory balance of covariates between groups (all SMD ≤0.1).

**TABLE 1:** Baseline characteristics with standardized mean differences (SMD) before and after inverse probability treatment weighting (IPTW).

	Metformin non‐users (*N* = 687)	Metformin users (*N* = 1284)	Unweighted SMD	SMD with IPTW
Age, years	78.3 ± 7.8	75.1 ± 7.7	0.42	0.08
Use of GnRH agonist or antagonist, *n* (%)	452 (65.8)	867 (67.5)	0.04	<0.01
Bilateral orchidectomy, *n* (%)	321 (46.7)	561 (43.7)	0.06	0.06
Hypertension, *n* (%)	415 (60.4)	580 (45.1)	0.31	0.09
Ischemic heart disease, *n* (%)	194 (28.2)	222 (17.3)	0.26	0.08
Myocardial infarction, *n* (%)	75 (10.9)	47 (3.7)	0.28	0.07
Heart failure, *n* (%)	108 (15.7)	84 (6.5)	0.29	0.09
Stroke or transient ischemic attack, *n* (%)	144 (21.0)	152 (11.8)	0.25	0.08
Chronic kidney disease, *n* (%)	109 (15.9)	35 (2.7)	0.46	0.12
Anemia, *n* (%)	108 (15.7)	111 (8.6)	0.22	0.07
Atrial fibrillation, *n* (%)	61 (8.9)	72 (5.6)	0.13	0.03
Chronic liver disease, *n* (%)	15 (2.2)	29 (2.3)	<0.01	0.02
Chronic obstructive pulmonary disease, *n* (%)	39 (5.7)	58 (4.5)	0.05	0.03
Hyperlipidemia, *n* (%)	170 (24.7)	267 (20.8)	0.09	0.02
Ever underwent radiotherapy, *n* (%)	116 (16.9)	238 (18.5)	0.04	0.02
Ever underwent radical prostatectomy, *n* (%)	233 (33.9)	405 (31.5)	0.05	<0.01
Any other malignancy, *n* (%)	104 (15.1)	151 (11.8)	0.10	0.03
ACEI/ARB use, *n* (%)	402 (58.5)	821 (63.9)	0.11	0.06
Beta‐blocker use, *n* (%)	403 (58.7)	571 (44.4)	0.29	0.08
Dihydropyridine calcium channel blocker use, *n* (%)	492 (71.6)	796 (61.9)	0.21	0.07
Non‐metformin anti‐diabetic medications, *n* (%)	449 (65.4)	919 (71.6)	0.14	0.04
Insulin use, *n* (%)	222 (32.3)	279 (21.7)	0.24	0.08
Statin use, *n* (%)	419 (61.0)	763 (59.4)	0.03	<0.01
Corticosteroid use, n (%)	153 (22.3)	23 (15.8)	0.17	0.06
Antiplatelet use, *n* (%)	335 (48.8)	442 (34.4)	0.30	0.07
Anticoagulant use, *n* (%)	50 (7.3)	65 (5.1)	0.09	0.02
Androgen receptor antagonist use, *n* (%)	257 (37.4)	579 (45.1)	0.15	0.10
Prior chemotherapy, *n* (%)	3 (0.4)	8 (0.6)	0.03	0.02
Chemotherapy concurrent with ADT, *n* (%)	36 (5.2)	129 (10.0)	0.18	0.06
HbA1c, %	6.7 ± 1.2	7.2 ± 1.3	0.34	0.08

*Note*: Mean ± standard deviation or frequency (count).

Abbreviations: ACEI, angiotensin converting enzyme inhibitor; ADT, androgen deprivation therapy; ARB, angiotensin receptor blocker; GnRH, gonadotropin hormone‐releasing hormone.

Over a total of 8,045 person‐years, 9,049 A&E attendances, 21,262 inpatient attendances, and 112,781 days of hospitalization were observed. The IR and IRR for each group, with metformin non‐users as reference, were summarized in Table 2. Metformin users had significantly fewer A&E attendances (IRR: 0.61 [0.54–0.69], *p* < 0.001), inpatient attendances (IRR: 0.57 [0.48–0.67], *p* < 0.001), and days of hospitalization (IRR: 0.55 [0.42–0.72], *p* < 0.001). These translated to lower annualized per‐patient A&E attendances‐related cost among metformin users (€193.21 [€180.64–€206.66] for metformin users, versus €314.70 [€283.58–€349.23] for metformin non‐users) as well as lower annualized per‐patient inpatient attendances‐related cost (€11,311.69 [€10,433.76–€12,263.49] for metformin users, versus €20,329.34 [€15,772.88–€26,202.07] for metformin non‐users). Overall, the estimated annualized per‐patient hospital attendances‐related cost was €12,111 [€11,235–€13,055] for metformin users and €42,952 [€15,184–€121,501] for non‐users, with a cost ratio of 0.28 [0.10–0.80] (*p* = 0.017), indicating that metformin users had lower hospital attendances‐related costs when compared to metformin non‐users. The sensitivity analysis using multivariable adjustments showed consistent results, where patients on metformin have significantly fewer hospital attendances and days of hospitalization compared to those not on metformin (all *p* < 0.001), with a cost ratio of 0.57 [0.50–0.66] (*p* < 0.001). These are summarized in Table S2.

**TABLE 2:** Annual incidence rates (IR) and ratios (IRR) of hospital attendances and days of hospitalization. IRRs were referenced against metformin non‐users.

	IR [95% confidence interval] of metformin users	IR [95% confidence interval] of metformin non‐users	IRR [95% confidence interval]	*p* Value
A&E	1.3 [1.2–1.4]	2.2 [2.0–2.4]	0.61 [0.54–0.69]	<0.001
Inpatient	3.0 [2.8–3.2]	5.4 [4.5–6.5]	0.57 [0.48–0.67]	<0.001
Days of hospitalization	18.4 [17.0–19.9]	33.8 [25.4–45.0]	0.55 [0.42–0.72]	<0.001

Abbreviation: A&E, accident and emergency.

These associations may be related to metformin's protective effects such that metformin was shown to lessen the development of ADT‐associated metabolic syndrome by decreasing systolic blood pressure and body mass index.^9^ Cost of management of cardiovascular events is often significantly higher than the cost of ADT itself.^10^ Our observations may therefore have been mediated by metformin preventing adverse cardiac events.

In addition to metformin's potential role in inhibiting the survival of PCa cells by androgen receptor signaling suppression,^11^ it may also delay metastatic disease mainly through adenosine monophosphate‐activated protein kinase‐dependent mechanisms.^12^ Since more hospital admissions, particularly inpatient, were observed among PCa patients with metastases,^13^ metformin use may be conducive in reducing needs for healthcare resources related to metastatic PCa. Future studies may investigate the potential effects of metformin in patients with PCa who did not receive ADT.

This study used a large, representative population‐based cohort with a long follow‐up period. Therefore, our results are likely widely applicable and reflect real‐world clinical practice. However, this study has a number of limitations, and should therefore be viewed as a hypothesis‐generating study which requires further, confirmatory work. Owing to its observational nature, unmeasured, or residual confounding may exist but was partly addressed by IPTW. Also, cancer staging is lacking; nonetheless, PCa treatments which may at least partially reflect disease severity were included as covariates in IPTW. In addition, patients whose diabetes is well controlled with metformin may have improved health maintenance and/or lower rates of diabetic comorbidities compared to non‐users; however, IPTW allowed for balancing and adjustment of these possible confounders. In addition, the data available could not be individually adjudicated; however, none of the authors had influence over data input.

Concurrent metformin use and ADT in Asian, diabetic adults with PCa was associated with decreased hospital attendances and the associated costs, regardless of the type of attendance.

## AUTHOR CONTRIBUTIONS

**Yan Hiu Athena Lee:** Conceptualization (lead); data curation (equal); formal analysis (equal); investigation (lead); methodology (lead); project administration (lead); validation (equal); visualization (equal); writing – original draft (equal). **Jeremy Man Ho Hui:** Conceptualization (lead); validation (equal); visualization (equal); writing – original draft (lead). **Cheuk To Chung:** Writing – original draft (equal). **Kang Liu:** Data curation (equal). **Edward Christopher Dee:** Writing – review and editing (equal). **Kenrick Ng:** Writing – review and editing (equal). **Jeffrey Shi Kai Chan:** Conceptualization (equal); formal analysis (equal); software (equal); validation (equal); writing – original draft (equal). **Chi Fai Ng:** Conceptualization (equal); supervision (equal); writing – review and editing (equal).

## CONFLICT OF INTEREST STATEMENT

The authors declare no conflict of interest.

## ETHICS APPROVAL

This study was approved by the Joint Chinese University of Hong Kong–New Territories East Cluster Clinical Research Ethics Committee.

## PATIENT CONSENT

Not applicable due to the nature of the study.

## PERMISSION TO REPRODUCE MATERIAL FROM OTHER SOURCES

Not applicable due to the nature of the study.

## CLINICAL TRIAL REGISTRATION

Not applicable due to the nature of the study.

## Supporting information


Figure S1.

Appendix S1.

## References

[1] Reichert ZR , Hussain M . Androgen receptor and beyond, targeting androgen signaling in castration‐resistant prostate cancer. Cancer J. 2016;22(5):326‐329. 27749325 10.1097/PPO.0000000000000214

[2] Gilbert SM , Kuo YF , Shahinian VB . Prevalent and incident use of androgen deprivation therapy among men with prostate cancer in the United States. Urol Oncol. 2011;29(6):647‐653. 19926311 10.1016/j.urolonc.2009.09.004 PMC2888908

[3] Hu JR , Duncan MS , Morgans AK , et al. Cardiovascular effects of androgen deprivation therapy in prostate cancer: contemporary meta‐analyses. Arterioscler Thromb Vasc Biol. 2020;40(3):e55‐e64. 31969015 10.1161/ATVBAHA.119.313046 PMC7047549

[4] Wallis CJD , Mahar AL , Cheung P , et al. Hospitalizations to manage complications of modern prostate cancer treatment in older men. Urology. 2016;96:142‐147. 27289026 10.1016/j.urology.2016.05.054

[5] Aboelnaga EM , Aboelnaga MM , Elkalla HM . Metformin addition to androgen deprivation therapy effect on cancer prostate patients with type 2 diabetes. Diabetes Metab Syndr. 2021;15(5):102251. 34428740 10.1016/j.dsx.2021.102251

[6] Ha Chung B , Horie S , Chiong E . The incidence, mortality, and risk factors of prostate cancer in Asian men. Prostate Int. 2019;7(1):1‐8. 30937291 10.1016/j.prnil.2018.11.001 PMC6424686

[7] Lee YHA , Hui JMH , Chan JSK , et al. Metformin use and mortality in Asian, diabetic patients with prostate cancer on androgen deprivation therapy: a population‐based study. Prostate. 2023;83(1):119‐127. 36178848 10.1002/pros.24443 PMC9742285

[8] Hospital Authority Ordinance (Chapter 113). 2020.

[9] Nobes JP , Langley SE , Klopper T , Russell‐Jones D , Laing RW . A prospective, randomized pilot study evaluating the effects of metformin and lifestyle intervention on patients with prostate cancer receiving androgen deprivation therapy. BJU Int. 2012;109(10):1495‐1502. 21933330 10.1111/j.1464-410X.2011.10555.x

[10] Krahn MD , Bremner KE , Luo J , Alibhai SM . Health care costs for prostate cancer patients receiving androgen deprivation therapy: treatment and adverse events. Curr Oncol. 2014;21(3):e457‐e465. 24940106 10.3747/co.21.1865 PMC4059810

[11] Akinyeke T , Matsumura S , Wang X , et al. Metformin targets c‐MYC oncogene to prevent prostate cancer. Carcinogenesis. 2013;34(12):2823‐2832. 24130167 10.1093/carcin/bgt307 PMC3845895

[12] Zaidi S , Gandhi J , Joshi G , Smith NL , Khan SA . The anticancer potential of metformin on prostate cancer. Prostate Cancer Prostatic Dis. 2019;22(3):351‐361. 30651580 10.1038/s41391-018-0085-2

[13] Tangirala K , Appukkuttan S , Simmons S . Costs and healthcare resource utilization associated with hospital admissions of patients with metastatic or nonmetastatic prostate cancer. Am Health Drug Benefits. 2019;12(6):306‐312. 31908714 PMC6922323

