# Supplementary figures and images for: Metformin use and hospital attendance‐related resources utilization among diabetic patients with prostate cancer on androgen deprivation therapy: A population‐based cohort study
Source: Cancer Med. 2023 Feb 3;12(8):9128–32. doi: 10.1002/cam4.5651 (PMC10166930; doi:10.1002/cam4.5651)

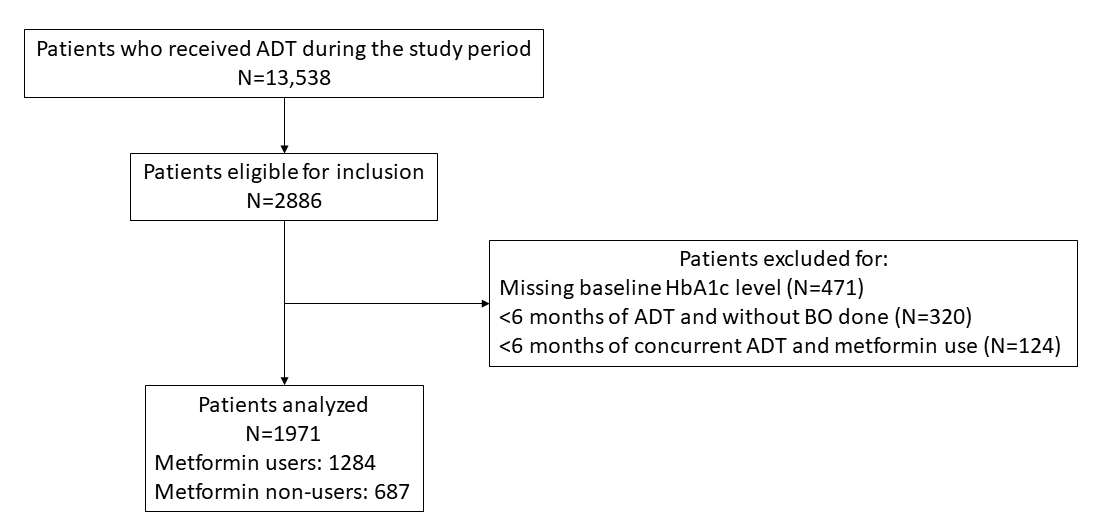

Supplement: Supplementary file 1 — Figure S1. [file CAM4-12-9128-s001.tif]
