# Supplementary material for: Metformin use and hospital attendance‐related resources utilization among diabetic patients with prostate cancer on androgen deprivation therapy: A population‐based cohort study
Source: Cancer Med. 2023 Feb 3;12(8):9128–32. doi: 10.1002/cam4.5651 (PMC10166930; doi:10.1002/cam4.5651)
Supplement: Supplementary file 2 — Appendix S1. [file CAM4-12-9128-s002.docx]

**Supplementary Table 1.** *International Classification of Diseases, Ninth Revision* codes used to identify outcomes and co-morbidities. All hereby listed codes include the corresponding sub-codes.

| Prostate cancer | 185 |
| --- | --- |
| Heart failure | 428 |
| Myocardial infarction | 410 |
| Diabetes mellitus | 250 |
| Hypertension | 401 402 403 404 405 437.2 |
| Atrial fibrillation | 427.31 |
| Stroke or transient ischemic attack | 430 431 432 433 434 435 |
| Chronic obstructive pulmonary disease | 490 491 492 496.0 |
| Ischemic heart disease | 410 411 412 413 414 |
| Chronic kidney disease | 582 585 586 |
| Chronic liver disease | 571 |
| Hyperlipidemia | 272.0 272.1 272.2 272.3 272.4 |
| Malignancy | 140 141 142 143 144 145 146 147 148 149 150 151 152 153 154 155 156 157 158 159 160 161 162 163 164 165 170 171 172 173 174 175 179 179 180 181 182 183 184 185 186 187 188 189 190 191 192 193 194 195 196 197 198 199 200 201 202 203 204 205 206 207 208 209.0 209.1 209.2 209.3 |

**Supplementary Table 2.** Results of the sensitivity analysis where multivariable adjustments was used instead of inverse probability treatment weighting to estimate the incidence rates ratios (IRR) of hospital attendances and days of hospitalization between groups. IRRs were referenced against metformin non-users.

|  | IRR [95% confidence interval] | p value |
| --- | --- | --- |
| A&E | 0.61 [0.55, 0.68] | <0.001 |
| Inpatient | 0.58 [0.52, 0.64] | <0.001 |
| Days of hospitalization | 0.59 [0.52, 0.66] | <0.001 |

A&E, accident and emergency.

Also: cost ratio on multivariable adjustment instead of IPTW: 0.57 [0.50, 0.66], p<0.001

**Supplementary Figure 1**. Study flow chart

ADT, androgen deprivation therapy. BO, bilateral orchidectomy.
